# Oral microbiome profiles by periodontitis stage in a Korean population

**DOI:** 10.3389/fcimb.2026.1809787

**Published:** 2026-04-27

**Authors:** Mu-Yeol Cho, Je-Hyun Eom, Ji-Won Kim, Yunwoo Kim, Jin-Ah Park, Hui-jeong Kim, Ju-Young Lee, Hye-lim Han, Se-Jeong Ko, Soo-Bok Her, Dong-Yub Ko, Hye-Sung Kim, Hanseung Baek

**Affiliations:** 1 Apple Tree Institute of Biomedical Science, Apple Tree Medical Foundation, Goyang-si, Republic of Korea; 2 Apple Tree Dental Hospital, Apple Tree Medical Foundation, Goyang-si, Republic of Korea; 3 Artificial Intelligence Research Center, Digital Dental Hub Incorporation, Seoul, Republic of Korea

**Keywords:** 16S rRNA sequencing, Korean population, microbial diversity, mouthwash, oral microbiome, periodontitis

## Abstract

**Background:**

Periodontitis is a chronic inflammatory disease driven by oral microbial dysbiosis. Although the oral microbiome has been characterized in diverse populations, comprehensive profiling across periodontal disease stages defined by the 2018 AAP/EFP classification remains limited in Korean adults.

**Methods:**

In this pilot prospective cross-sectional study, oral microbiome profiles were characterized in 74 participants classified into three groups: healthy controls (*n* = 24), Stage I–II periodontitis (*n* = 12), and Stage III–IV periodontitis (*n* = 38). Mouthwash samples were collected and subjected to 16S rRNA gene sequencing of the V3–V4 hypervariable region. Alpha diversity, beta diversity (PERMANOVA with sequential covariate adjustment for age, sex, and smoking), differential abundance (MaAsLin2), and core microbiome analyses were performed.

**Results:**

Stage III–IV periodontitis was associated with significantly higher Shannon diversity, Simpson diversity, and Pielou’s evenness compared to both healthy and Stage I–II groups, indicating increased evenness rather than species richness. Beta diversity analyses revealed significant community-level separation across groups after adjustment for demographic confounders (all*p* = 0.001). Differential abundance analysis identified 14 genera significantly associated with disease status. Twelve genera were enriched in Stage III–IV, including established periodontal pathogens Tannerella and Treponema, as well as emerging pathobionts Filifactor and Fretibacterium. Rothia and Kingella were enriched in periodontal health, consistent with their roles in nitrate reduction and maintenance of a health-compatible oral environment. Core microbiome analysis identified 40 universally present genera, with Anaeroglobus detected exclusively in Stage III–IV at 100% prevalence.

**Conclusion:**

These findings support the polymicrobial synergy and dysbiosis model of periodontitis pathogenesis and provide a foundation for developing microbiome-based diagnostic tools for periodontal disease assessment in Korean populations.

## 1 Introduction

Periodontitis is a chronic inflammatory disease characterized by progressive destruction of tooth-supporting tissues, affecting approximately 50% of the global adult population (Kassebaum et al., 2014). The oral microbiome plays a central role in periodontal health and disease, with dysbiotic shifts from commensal-dominated communities toward pathogenic consortia driving disease progression (Hajishengallis and Lamont, 2012). While the classical red complex bacteria have long been recognized as key pathogens, recent sequencing advances have revealed additional species contributing to the polymicrobial nature of periodontitis (Socransky et al., 1998; Pérez-Chaparro et al., 2014).

The composition of the oral microbiome is influenced not only by disease status but also by host factors including ethnicity, diet, and geographic origin. Studies have demonstrated significant differences in oral microbial diversity and community composition across racial and ethnic groups, with several periodontitis-associated taxa showing differential prevalence by ancestry (Yang et al., 2019). Although preliminary microbiome characterization in Korean periodontitis patients has been reported (Ko et al., 2020; Na et al., 2020), comprehensive profiling across discrete periodontal disease stages defined by the 2018 AAP/EFP classification system has not been established in this population.

Most periodontal microbiome studies have focused on subgingival plaque samples, which require invasive collection procedures. Mouthwash sampling offers a non-invasive alternative that integrates microbial communities from multiple oral niches, including supragingival plaque, buccal mucosa, and saliva, making it particularly suitable for population-level studies (Belstrøm, 2020). While this approach may not fully reflect the localized subgingival environment, it provides a representative snapshot of the overall oral microbial reservoir and has been validated as a sampling method for oral microbiome studies (Yano et al., 2023).

Therefore, this study aimed to characterize oral microbiome profiles across periodontal health and disease stages in Korean adults using 16S rRNA gene sequencing of mouthwash samples, investigating microbial diversity, differentially abundant taxa, and core microbiome features associated with disease progression.

## 2 Materials and methods

### 2.1 Study design and ethical approval

This pilot prospective cross-sectional study was conducted at Appletree Dental Hospital (Goyang, Republic of Korea). The study protocol was approved by the Institutional Review Board of Appletree Medical Foundation (IRB No. ATDH-2025-0001) and registered in the Clinical Research Information Service (CRIS, KCT0011511). All procedures were performed in accordance with the Declaration of Helsinki, and written informed consent was obtained from all participants prior to enrollment.

### 2.2 Study population

Participants were recruited from patients visiting Appletree Dental Hospital between February and July 2025. Periodontal status was classified according to the 2018 American Academy of Periodontology classification system (Tonetti et al., 2018). Adults aged 19 years or older were eligible for inclusion. Participants in the healthy group were required to have clinically healthy periodontal tissue with probing depth of 3 mm or less at all sites and bleeding on probing in fewer than 10% of sites. Those in the periodontitis group were diagnosed with Stage I through IV periodontitis based on interdental clinical attachment loss, radiographic bone loss, and tooth loss attributable to periodontitis.

Participants were excluded if they had used anti-inflammatory, immunosuppressive medications, or antibiotics within the past 3 months, received periodontal treatment including scaling and root planting within the past 3 months, had uncontrolled systemic diseases, were pregnant, had cardiovascular, respiratory, renal, hepatic, gastrointestinal, hematological, neuropsychiatric, or thyroid disorders, a history of oral malignancy, other oral inflammatory conditions, or a history of substance abuse within the past year.

### 2.3 Clinical examination

Clinical periodontal parameters including probing depth and bleeding on probing were recorded by three skilled examiners at six sites per tooth. Panoramic radiographs were analyzed using an artificial intelligence-based platform (PANO; DDH Co., Ltd., Korea) to measure interdental crestal bone level from the cementoenamel junction, similar to previous deep learning approaches for periodontal bone loss assessment (Chang et al., 2020). Estimated clinical attachment loss (eCAL) was calculated by subtracting the biological width of 2.04 mm from the measured crestal bone level, based on histological reference values (Gargiulo et al., 1961).

### 2.4 Sample collection

Prior to sample collection, all participants performed tooth brushing using identical toothbrushes and toothpaste provided by the research team, to standardize pre-sampling oral hygiene conditions and minimize interindividual variability in residual microbial load attributable to recent dietary intake or habitual oral hygiene practices. Oral rinse samples were collected using 10 mL of COOL SENSE mouthwash solution (Docsmedi Co., Ltd., Korea). Participants were instructed to swish the solution vigorously between teeth for 30 seconds without tilting the head backward, then expectorate into a sterile collection tube. Samples were refrigerated immediately after collection and stored at −80 °C until analysis.

### 2.5 16S rRNA gene sequencing

Genomic DNA was extracted from oral rinse samples using the FastDNA Spin Kit for Soil (MP Biomedicals, USA). DNA concentration was measured using an Epoch™ Spectrometer (BioTek, USA), and DNA integrity was confirmed by 1% agarose gel electrophoresis. The V3–V4 hypervariable region of the 16S rRNA gene was amplified using the primer pair 341F (5′-CCTACGGGNGGCWGCAG-3′) and 805R (5′-GACTACHVGGGTATCTAATCC-3′). PCR amplification was performed using TaKaRa Ex Taq DNA polymerase (TaKaRa Bio, Japan) under the following conditions: initial denaturation at 95 °C for 3 min; 25 cycles of 95 °C for 30 s, 55 °C for 30 s, and 72 °C for 30 s; and a final extension at 72 °C for 5 min. Index PCR was subsequently performed for 8 cycles using Nextera XT index primers. Library quality was assessed using the Agilent 2100 Bioanalyzer System (Agilent Technologies, USA) and quantified using the Quant-iT PicoGreen dsDNA Assay Kit (Invitrogen, USA). Paired-end sequencing (2 × 250 bp) was performed on the Illumina MiSeq platform using the MiSeq Reagent Kit v2 at CJ Bioscience (Seoul, Korea). Raw sequencing data were processed using the EzBioCloud pipeline, which included quality filtering, chimera removal using UCHIME, and taxonomic assignment against the EzBioCloud database (PKSSU4.0) at 97% sequence similarity for species-level identification (Yoon et al., 2017). Raw sequencing data have been deposited in the NCBI Sequence Read Archive (SRA) under BioProject accession number PRJNA1422962.

### 2.6 Statistical analysis

Taxonomic composition data expressed as relative abundance were used for downstream analyses. Alpha diversity indices including Chao1, Shannon, Simpson, and Pielou’s evenness were calculated, and differences among groups were evaluated using the Kruskal-Wallis test with Dunn’s *post-hoc* test. Beta diversity was assessed using Bray-Curtis dissimilarity, Generalized UniFrac, and UniFrac. To account for potential confounding effects, PERMANOVA was conducted using a sequential (Type I) model via the adonis2 function in the vegan package (R), with age, sex, and smoking status entered as covariates prior to periodontal group. Unadjusted models were also computed for comparison. All PERMANOVA analyses were performed with 999 permutations. Rarefaction was not applied, as sequencing depth did not differ significantly among groups (Kruskal–Wallis test,*p* = 0.324; **Supplementary Figure 1**). Differential abundance analysis was performed using MaAsLin2 (Multivariable Association Discovery in Population-scale Meta-omics Studies), with age, sex, and smoking status included as covariates to adjust for potential confounding effects. Taxa with adjusted *q* < 0.05 (Benjamini-Hochberg correction) and absolute MaAsLin2 coefficient > 0.1 were considered differentially abundant. To minimize spurious findings, only taxa present in at least 30% of samples with mean relative abundance of at least 0.1% were included in the analysis. Core microbiome was defined as genera present in 100% of samples within each group. This stringent threshold was selected to identify taxa universally associated with each periodontal status (Zaura et al., 2009). Statistical analyses were performed using R software (version 4.5.1). A two-tailed *p*-value < 0.05 was considered statistically significant.

## 3 Results

### 3.1 Study population characteristics

Of the 104 initially recruited participants, 30 were excluded due to gingivitis diagnosis not meeting classification criteria (*n* = 5), recent antibiotic use, dental treatment history, or incomplete data (*n* = 25). The final analysis included 74 participants: 24 healthy controls, 12 with Stage I–II, and 38 with Stage III–IV (**Table 1**). The three groups differed significantly in age, sex distribution, and smoking status. Mean age increased progressively from healthy (29.42 ± 5.89 years) to Stage I–II (45.92 ± 11.59 years) to Stage III–IV (62.18 ± 9.05 years), with all pairwise comparisons statistically significant (*p* < 0.001). The healthy group was predominantly female (87.5%), whereas males comprised over half of both periodontitis groups (*p* < 0.001). Current smokers were absent in the healthy group and present in 33.3% of Stage I–II and 15.8% of Stage III–IV (*p* = 0.013).

**Table 1:** Demographic information of the study subjects.

Variable	Healthy (*n* = 24)	Stage I–II (*n* = 12)	Stage III–IV (*n* = 38)	*p*-value	Bonferroni *post-hoc*
Age	29.42 ± 5.89	45.92 ± 11.59	62.18 ± 9.05	<0.001^a^	H < I**–**II, H < III**–**IV, I**–**II < III**–**IV
Age group, *n* (%)					
20–39 yrs	22 (91.67%)	3 (25.00%)	0 (0.00%)	<0.001^b^	
40–59 yrs	2 (8.33%)	8 (66.67%)	13 (34.21%)		
Over 60 yrs	0 (0.00%)	1 (8.33%)	25 (65.79%)		
Sex, *n* (%)					
Male	3 (12.50%)	8 (66.67%)	21 (55.26%)	<0.001^b^	
Female	21 (87.50%)	4 (33.33%)	17 (44.74%)		
Smoking status, *n* (%)					
No (nonsmoker)	24 (100.00%)	8 (66.67%)	32 (84.21%)	0.013^b^	
Yes (current smoker)	0 (0.00%)	4 (33.33%)	6 (15.79%)		
Clinical parameters					
Total n of teeth	27.75 ± 1.03	27.50 ± 1.88	18.32 ± 7.25	<0.001^a^	III**–**IV < H, III**–**IV < I**–**II
Mean PD (mm)	2.18 ± 0.54	2.73 ± 0.37	3.53 ± 0.92	<0.001^a^	H < III**–**IV, I**–**II < III**–**IV
Max PD (mm)	3.00 ± 0.00	4.83 ± 0.94	8.37 ± 1.79	<0.001^a^	H < I**–**II, H < III**–**IV, I**–**II < III**–**IV
BOP (%)	2.96 ± 2.92	14.07 ± 9.89	37.58 ± 30.72	<0.001^a^	H < III**–**IV, I**–**II < III**–**IV
Mean eCAL (mm)	0.36 ± 0.16	0.98 ± 0.56	2.92 ± 0.79	<0.001^a^	H < I**–**II, H < III**–**IV, I**–**II < III**–**IV
Max eCAL (mm)	1.85 ± 0.60	2.82 ± 0.98	6.93 ± 1.71	<0.001^a^	H < III**–**IV, I**–**II < III**–**IV
Oral hygiene habits					
Brushing frequency (times/day)	2.92 ± 1.44	2.25 ± 0.62	2.45 ± 0.69	0.099	
Floss (days/week)	3.26 ± 2.65	1.50 ± 2.68	1.00 ± 2.15	0.003^a^	III**–**IV < H
Interdental brush (days/week)	0.95 ± 2.16	3.64 ± 2.50	3.54 ± 3.08	0.003^a^	H < I**–**II, H < III**–**IV
Mouthwash (days/week)	2.05 ± 2.74	1.83 ± 2.37	2.43 ± 2.91	0.771	

Values are presented as mean ± standard deviation or *n* (%).

n, number; H, healthy; I**–**II, stage I**–**II; III**–**IV, stage III**–**IV; PD, probing depth; BOP, bleeding on probing; eCAL, estimated clinical attachment loss.

^a^ Statistically significant among groups by ANOVA (*p* < 0.05).

^b^ Statistically significant among groups by Chi-square test (*p* < 0.05).

All clinical periodontal parameters including mean probing depth, bleeding on probing, and estimated clinical attachment loss increased progressively with disease severity (all*p* < 0.001). The number of remaining teeth was lowest in Stage III–IV (18.32 ± 7.25), approximately one-third fewer than healthy controls (27.75 ± 1.03;*p* < 0.001). Dental floss use was highest in healthy participants (3.26 days/week) and lowest in Stage III–IV (1.00 days/week;*p* = 0.003). Conversely, interdental brush use was higher in periodontitis groups (Stage I–II: 3.64; Stage III–IV: 3.54 days/week) compared to healthy controls (0.95 days/week;*p* = 0.003).

### 3.2 Sequencing data and microbial diversity

**Figure 1:**
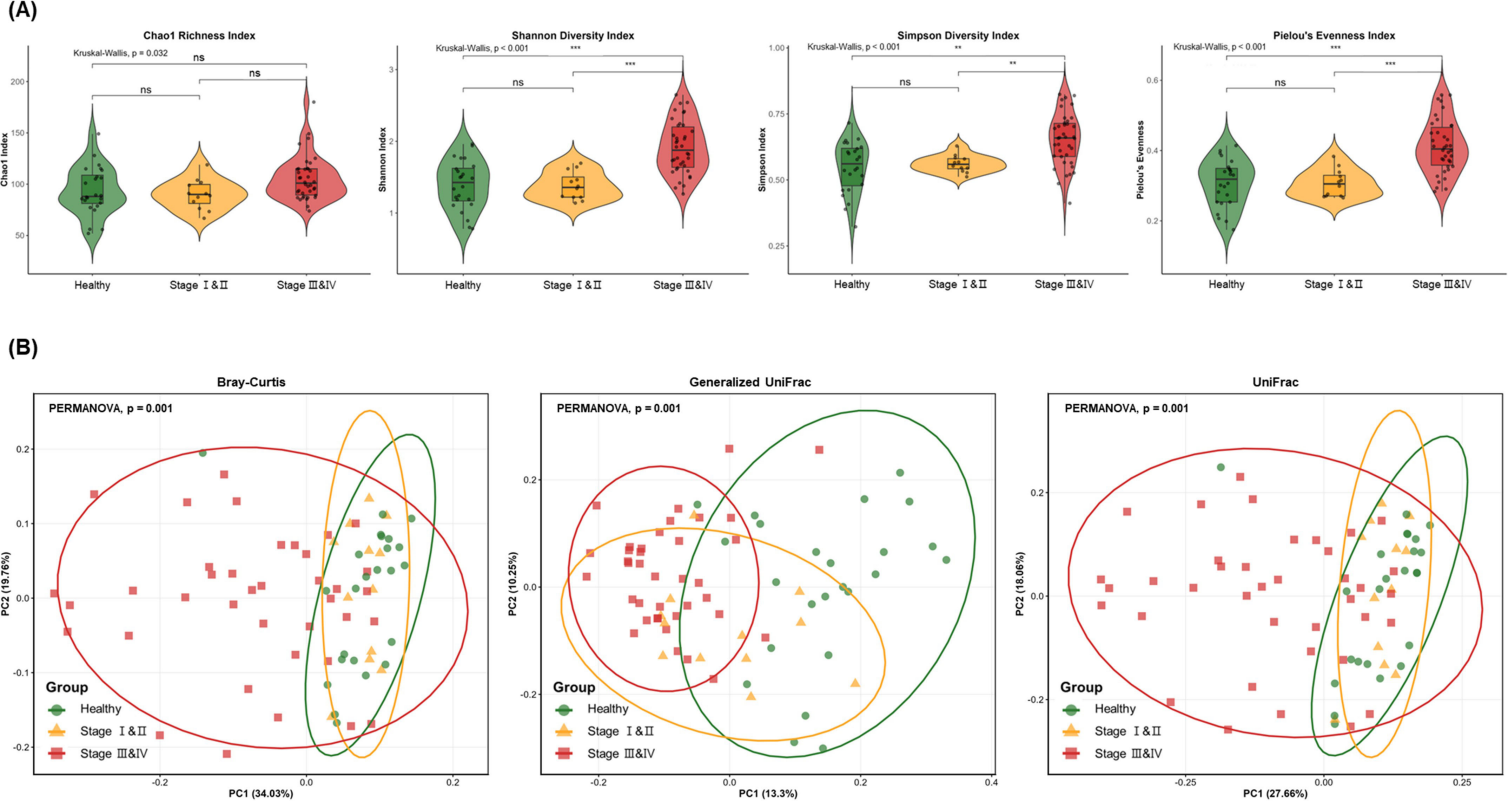
Diversity analysis of oral microbiome among healthy, stage I–II, and stage III–IV periodontitis groups. **(A)** Violin plots with box plots of alpha diversity indices including Chao1 richness, Shannon diversity, Simpson diversity, and Pielou’s evenness. **(B)** Principal Coordinate Analysis plots of beta diversity based on Bray-Curtis dissimilarity, Generalized UniFrac, and UniFrac distances. Ellipses indicate 95% confidence intervals. *p < 0.05, **p < 0.01, ***p < 0.001; ns, not significant (Kruskal-Wallis with pairwise Wilcoxon test for alpha diversity; PERMANOVA for beta diversity).

A total of 18,751,818 quality-filtered reads were obtained from 74 samples, with an average of 253,403 reads per sample. Sequencing depth did not differ significantly among groups (Kruskal–Wallis test,*p* = 0.324; **Supplementary Figure 1**), supporting the comparability of diversity analyses without rarefaction. Taxonomic classification identified 8,740 operational taxonomic units assigned to 28 phyla and 449 genera. Among alpha diversity indices, Shannon diversity, Simpson diversity, and Pielou’s evenness were significantly higher in Stage III–IV compared to both healthy and Stage I–II groups (*p* < 0.001), whereas healthy and Stage I–II groups did not differ from each other (**Figure 1A**). Species richness (Chao1) showed no significant pairwise differences after correction. Beta diversity analysis revealed distinct microbial community structures based on disease status (**Figure 1B**). After sequential adjustment for age, sex, and smoking, the effect of periodontal group remained significant across all three distance metrics (Bray-Curtis, Generalized UniFrac, and UniFrac; all*p* = 0.001; **Supplementary Table 1**), confirming that community differences were not attributable to demographic confounders. Stage III–IV was clearly separated from both healthy and Stage I–II groups across all three distance metrics. By contrast, healthy and Stage I–II groups showed no significant difference by any of the three metrics (Bray-Curtis:*p* = 0.104; Generalized UniFrac: *p* = 0.160; UniFrac:*p* = 0.137; **Supplementary Table 2**), suggesting that early-stage periodontitis is not yet associated with detectable community-level shifts relative to health.

### 3.3 Taxonomic composition

**Figure 2:**
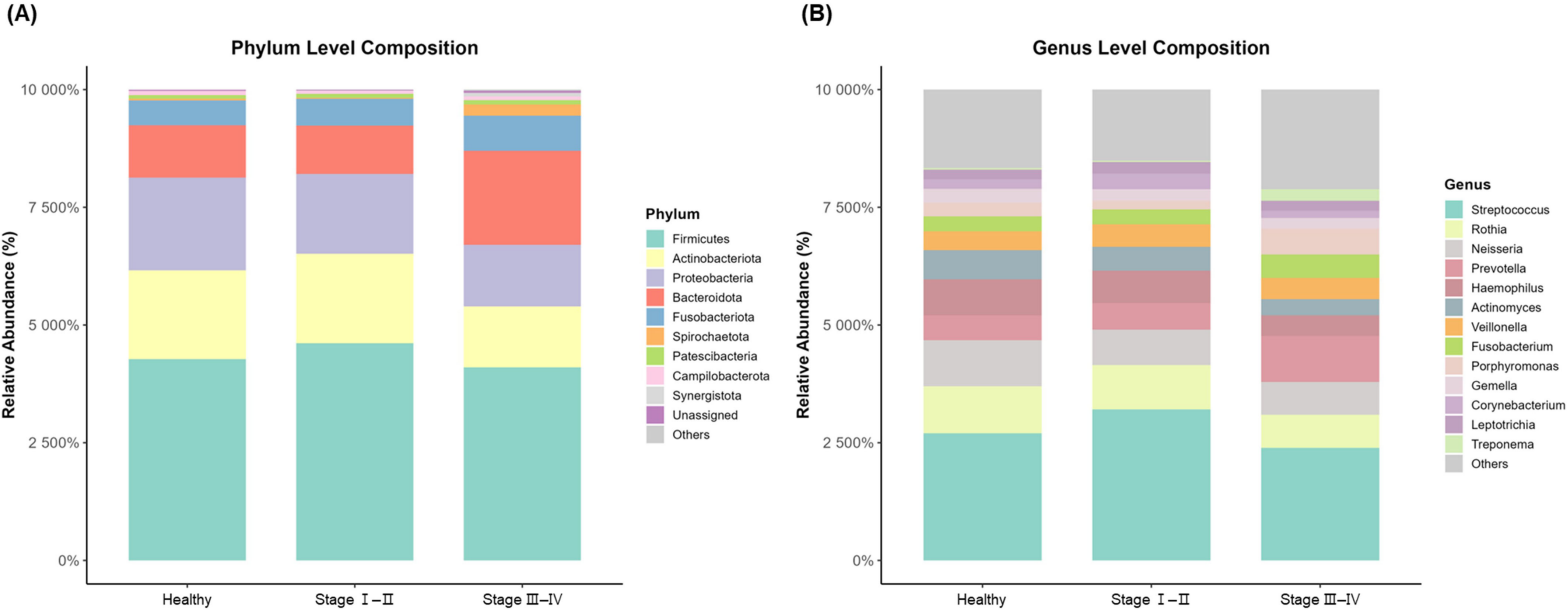
Taxonomic composition of oral microbiome across periodontal health status groups. Stacked bar plots of relative abundance at phylum level **(A)** and genus level **(B)**.

At the phylum level, Firmicutes dominated all groups, followed by Actinobacteriota, Proteobacteria, and Bacteroidota (**Figure 2A**). Bacteroidota nearly doubled in Stage III–IV compared to healthy controls, and Spirochaetota increased 7-fold in Stage III–IV. At the genus level, *Streptococcus* was dominant regardless of disease status (**Figure 2B**). *Prevotella*, *Porphyromonas*, and *Fusobacterium* increased with disease severity, with *Treponema* showing the most dramatic change (7-fold increase in Stage III–IV vs. healthy). Conversely, *Rothia*, *Haemophilus*, and *Actinomyces* decreased with disease progression. Detailed taxonomic composition at the phylum and genus levels is provided in **Supplementary Tables 3, S4**.

### 3.4 Differentially abundant taxa

**Figure 3:**
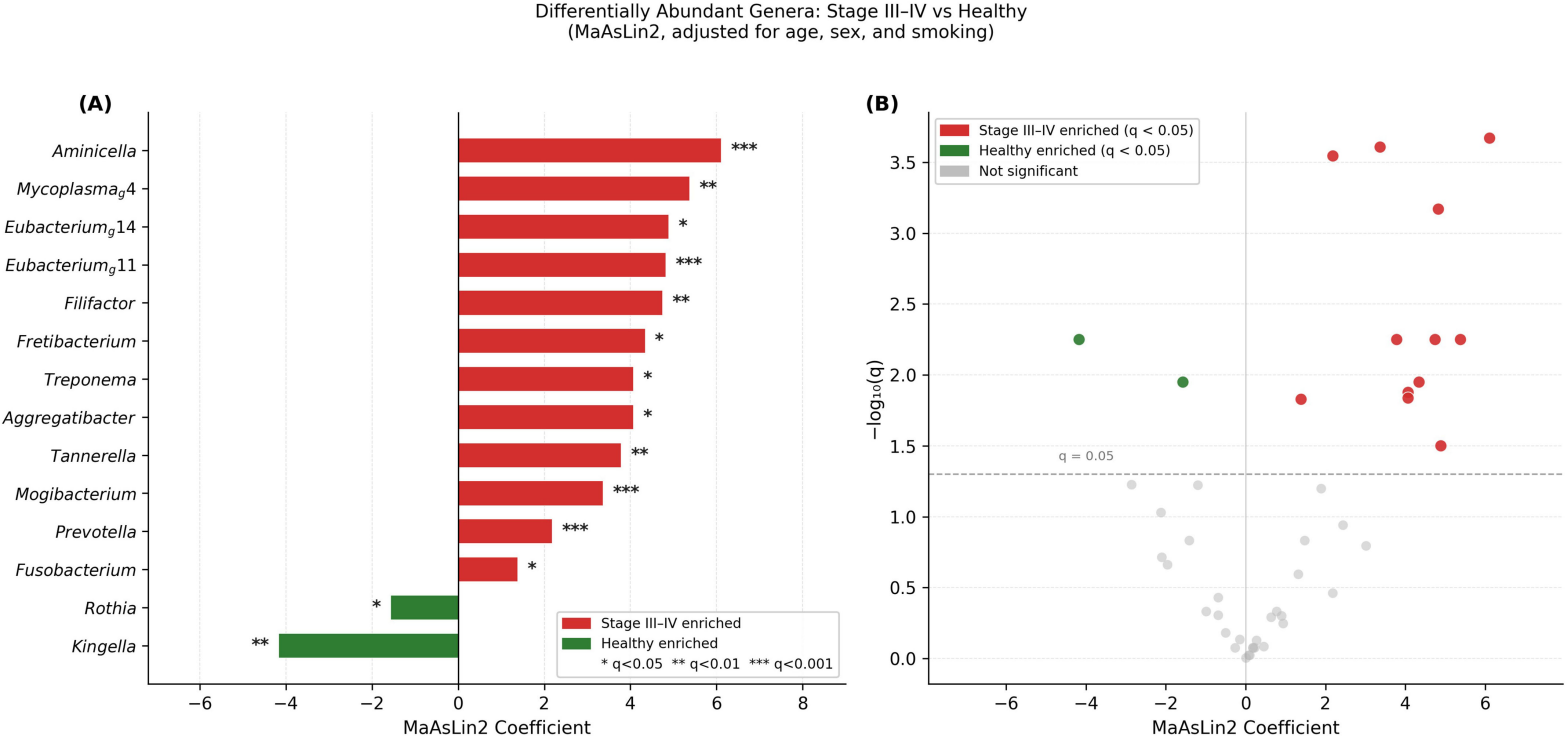
Differentially abundant genera between stage III–IV periodontitis and healthy controls. **(A)** Bar plot showing MaAsLin2 coefficients for genera significantly associated with periodontal disease stage after adjustment for age, sex, and smoking status. Positive and negative coefficients indicate enrichment in Stage III–IV and healthy groups, respectively. Significance: * *q* < 0.05, ** *q* < 0.01, *** *q* < 0.001 (Benjamini–Hochberg correction). **(B)** Volcano plot displaying all 44 tested genera. The x-axis represents the MaAsLin2 coefficient; the y-axis represents −log_10_(*q*-value). The dashed line indicates *q* = 0.05. Red and green dots indicate genera significantly enriched in Stage III–IV and healthy groups, respectively; grey dots indicate non-significant genera.

Differential abundance analysis identified 14 genera with significant differences between Stage III–IV and healthy groups after covariate adjustment (**Figure 3**). Twelve genera were enriched in Stage III–IV, with *Aminicella* showing the largest effect (coefficient = 6.111), followed by *Mycoplasma* g4, *Eubacterium* g14, *Eubacterium* g11, *Filifactor*, *Fretibacterium*, *Treponema*, *Aggregatibacter*, *Tannerella*, *Mogibacterium*, *Prevotella*, and *Fusobacterium* (**Figure 3A**). Two genera, *Kingella* and *Rothia*, were enriched in healthy controls. Volcano plots of all tested genera are presented in **Figure 3B**. Complete differential abundance results including MaAsLin2 coefficients, standard errors, and FDR-adjusted *q*-values are presented in **Supplementary Table 5**.

### 3.5 Core microbiome

**Figure 4:**
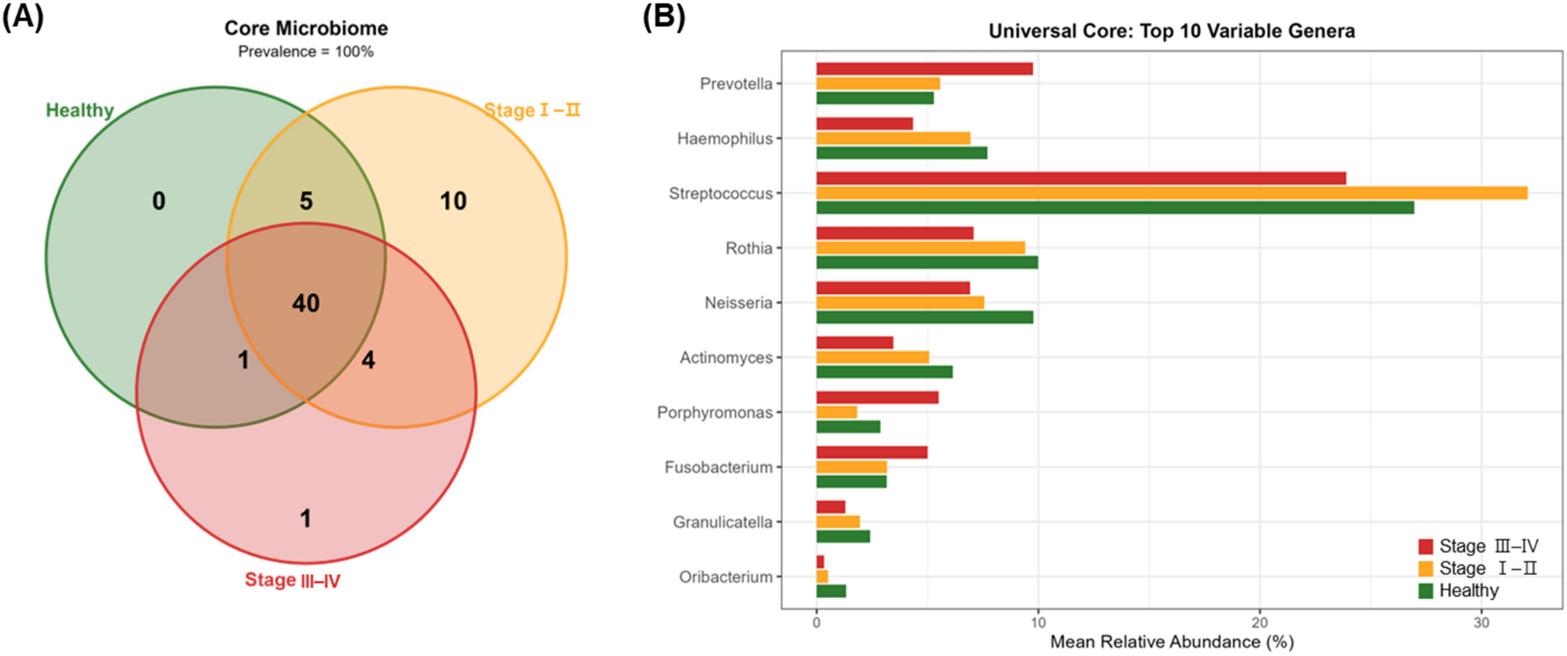
Core microbiome composition across periodontal health status. **(A)** Venn diagram showing the distribution of core microbiome genera (100% prevalence within each group) among periodontal health status groups. Numbers indicate the count of genera shared or unique to each group. **(B)** Mean relative abundance of top 10 universal core genera showing the largest abundance changes between Healthy and Stage III**–**IV groups. These genera were detected in 100% of samples across all three groups.

Core microbiome analysis identified 40 genera present in 100% of samples across all three groups (**Figure 4A**). The healthy group harbored 0 unique core genera, Stage I–II had 10 unique core genera, and Stage III–IV contained 1 unique core genus (*Anaeroglobus*). Among the 40 universal core genera, the top 10 showing the largest abundance changes between healthy and Stage III–IV groups are shown in **Figure 4B**. The full list of core genera with prevalence and abundance data is provided in **Supplementary Table 6**.

## 4 Discussion

This study characterized the oral microbiome across periodontal health and disease stages in a Korean adult population using 16S rRNA gene sequencing. Our findings demonstrated that Stage III–IV was associated with significantly higher microbial diversity, distinct community composition, and enrichment of both established and emerging periodontal pathogens. These results validate established periodontal disease paradigms while providing comprehensive microbiome profiles in a Korean population.

Alpha diversity analysis revealed that Shannon, Simpson, and Pielou’s evenness indices were significantly higher in the Stage III–IV group compared to healthy controls, whereas Chao1 index showed no significant difference between groups. This pattern indicates that the increased diversity is primarily driven by greater evenness rather than species richness. Such a shift from commensal-dominated communities toward a more even distribution of multiple pathogenic taxa is consistent with the polymicrobial synergy and dysbiosis model (Hajishengallis and Lamont, 2012). These findings contrast with previous subgingival plaque studies reporting decreased alpha diversity in periodontitis (Nibali et al., 2022). This discrepancy may be attributed to differences in sampling sites. Subgingival plaque reflects the localized anaerobic environment of periodontal pockets, whereas mouthwash samples represent the overall oral microbial reservoir. Consistent with our findings, Na et al. demonstrated higher microbial diversity in the buccal mucosa and supragingival plaque of Korean periodontitis patients (Na et al., 2020). Similarly, a recent study using full-length 16S rRNA sequencing reported elevated diversity in supragingival samples from periodontitis subjects (Wen et al., 2025). Beta diversity analysis revealed significant community-level separation between groups. This finding aligns with previous studies demonstrating distinct microbial community structures between periodontitis and healthy subjects (Soueidan et al., 2024).

Differential abundance analysis identified 14 genera with significant differences between Stage III–IV and healthy groups. Of these, 12 genera were enriched in Stage III–IV, including established periodontal pathogens such as *Tannerella* and *Treponema*, which constitute key members of the classical red complex associated with severe periodontitis (Socransky et al., 1998). *Treponema*, showing marked enrichment in Stage III–IV, is a highly proteolytic spirochete with documented virulence factors including dentilisin and outer membrane proteins that contribute to tissue destruction (Dashper et al., 2011). *Filifactor* is increasingly recognized as an emerging periodontal pathogen with unique characteristics including oxidative stress resistance and the ability to modulate host immune responses (Aja et al., 2021). Similarly, *Fretibacterium*, a member of the phylum Synergistetes, has been consistently associated with periodontal disease severity and proposed as a novel biomarker for periodontitis (Vartoukian et al., 2013; Pérez-Chaparro et al., 2014). Notably, *Aminicella* exhibited the largest effect size among all differentially abundant genera; however, as this genus has limited precedent in oral microbiome research (Ko et al., 2020), its detection may reflect environmental or reagent-derived contamination rather than a true biological signal, and should be interpreted with caution pending further validation. Conversely, *Rothia* and *Kingella* were enriched in healthy controls. *Rothia* species are recognized as commensals that contribute to nitrate reduction and maintenance of a health-compatible oral environment (Rosier et al., 2024), and their depletion in periodontitis has been reported in previous studies (Chen et al., 2018). Kingella, also enriched in healthy controls, is similarly recognized as a health-associated oral commensal capable of oral nitrate reduction (Barbagallo et al., 2022), and its co-depletion with Rothia in periodontitis further supports the concept of impaired nitrate-reducing capacity as a feature of periodontal dysbiosis.

Core microbiome analysis revealed 40 genera that were universally present across all groups, representing a stable microbial foundation that persists regardless of disease status. This finding aligns with previous studies demonstrating that a substantial portion of oral taxa are shared between health and periodontitis, supporting the concept of a conserved core microbiome (Zaura et al., 2009; Abusleme et al., 2013). Notably, only one genus, *Anaeroglobus*, was uniquely reaching 100% prevalence in the core microbiome of Stage III–IV. This finding is consistent with previous reports identifying *Anaeroglobus geminatus* as a putative periodontal pathogen that promotes the growth of other pathogenic species and enhances biofilm virulence (Bao et al., 2017), and its exclusive detection in severe periodontitis (Plachokova et al., 2021). In contrast, the healthy group harbored no group-exclusive core genus, suggesting that periodontal dysbiosis is driven by proportional shifts within a shared microbial pool rather than acquisition of entirely novel taxa, consistent with the ecological succession model of periodontitis.

This study has several limitations. First, the cross-sectional design precludes establishing causal relationships between microbiome changes and periodontal disease progression. Second, the pronounced sex imbalance across groups may represent residual confounding despite inclusion of sex as a covariate in all multivariate analyses. Third, mouthwash sampling captures the overall oral microbiome but may not fully represent site-specific subgingival communities where periodontal pathogens predominantly reside. Pre-sampling tooth brushing was standardized across participants; however, its mechanical effects on residual microbiota may vary with periodontal pocket depth, and the use of a commercially available antiseptic mouthwash (COOL SENSE; Docsmedi Co., Ltd., Korea) rather than sterile saline may have had selective effects on specific taxa that cannot be entirely excluded. Fourth, the Stage I–II group comprised only 12 participants, which may have limited statistical power to detect inter-group differences; results for this group should be interpreted with caution. Fifth, inter-examiner reliability for clinical periodontal measurements was not formally assessed, and the absence of reported Kappa values represents a methodological limitation. Additionally, gingival recession was not measured, precluding traditional calculation of clinical attachment loss from probing depth and recession. Instead, attachment loss was estimated using AI-based panoramic radiographic analysis. A recent study has demonstrated that AI-based panoramic analysis achieves accuracy (90.4%) comparable to traditional radiographic assessment by trained examiners (85.7%) (Kao et al., 2025). Nevertheless, panoramic radiographs are inherently subject to magnification and distortion compared to periapical radiographs, and the application of a fixed biological width value may introduce systematic estimation error; the potential for disease stage misclassification cannot be entirely excluded. Finally, while 16S rRNA sequencing provides valuable taxonomic information, it does not capture species-level resolution within clinically relevant genera or functional and virulence characteristics of the detected taxa. Future studies incorporating full-length 16S sequencing, metagenomics, or metatranscriptomics approaches could provide deeper insights into the functional roles of oral microbiota in periodontitis pathogenesis.

In conclusion, this study characterized oral microbiome profiles across periodontal health and disease stages using 16S rRNA sequencing of mouthwash samples. Stage III–IV was associated with significantly higher microbial diversity and distinct community composition compared to periodontal health. We identified 14 differentially abundant genera, with enrichment of established pathogens including *Tannerella*, *Treponema*, and *Filifactor*, as well as emerging pathobionts such as *Fretibacterium* in Stage III–IV periodontitis. Conversely, *Rothia* and *Kingella* were enriched in periodontal health, consistent with their roles in oral nitrate reduction and maintenance of a health-compatible microbial environment. The exclusive detection of *Anaeroglobus* in Stage III–IV periodontitis warrants further investigation into its potential utility as a disease-stage-specific indicator. These findings support the polymicrobial synergy and dysbiosis model of periodontitis pathogenesis and provide a foundation for developing microbiome-based diagnostic tools for periodontal disease assessment.

Box plots showing the number of valid reads per sample in each group. Boxes represent the interquartile range; horizontal lines indicate medians; whiskers extend to 1.5× the interquartile range. Individual data points are overlaid. No significant difference in sequencing depth was observed among groups (Kruskal–Wallis test,*p* = 0.324), supporting the comparability of diversity analyses without rarefaction.

## Data Availability

The 16S rRNA gene sequencing data generated in this study have been deposited in the NCBI Sequence Read Archive (SRA) under BioProject accession number PRJNA1422962.

## Supplementary material

Supplementary Figure 1 Sequencing depth distribution across periodontal status groups.
