# Supplementary figures and images for: Oral microbiome profiles by periodontitis stage in a Korean population
Source: Front Cell Infect Microbiol. 2026 Apr 27;16:1809787. doi: 10.3389/fcimb.2026.1809787 (PMC13158189; doi:10.3389/fcimb.2026.1809787)

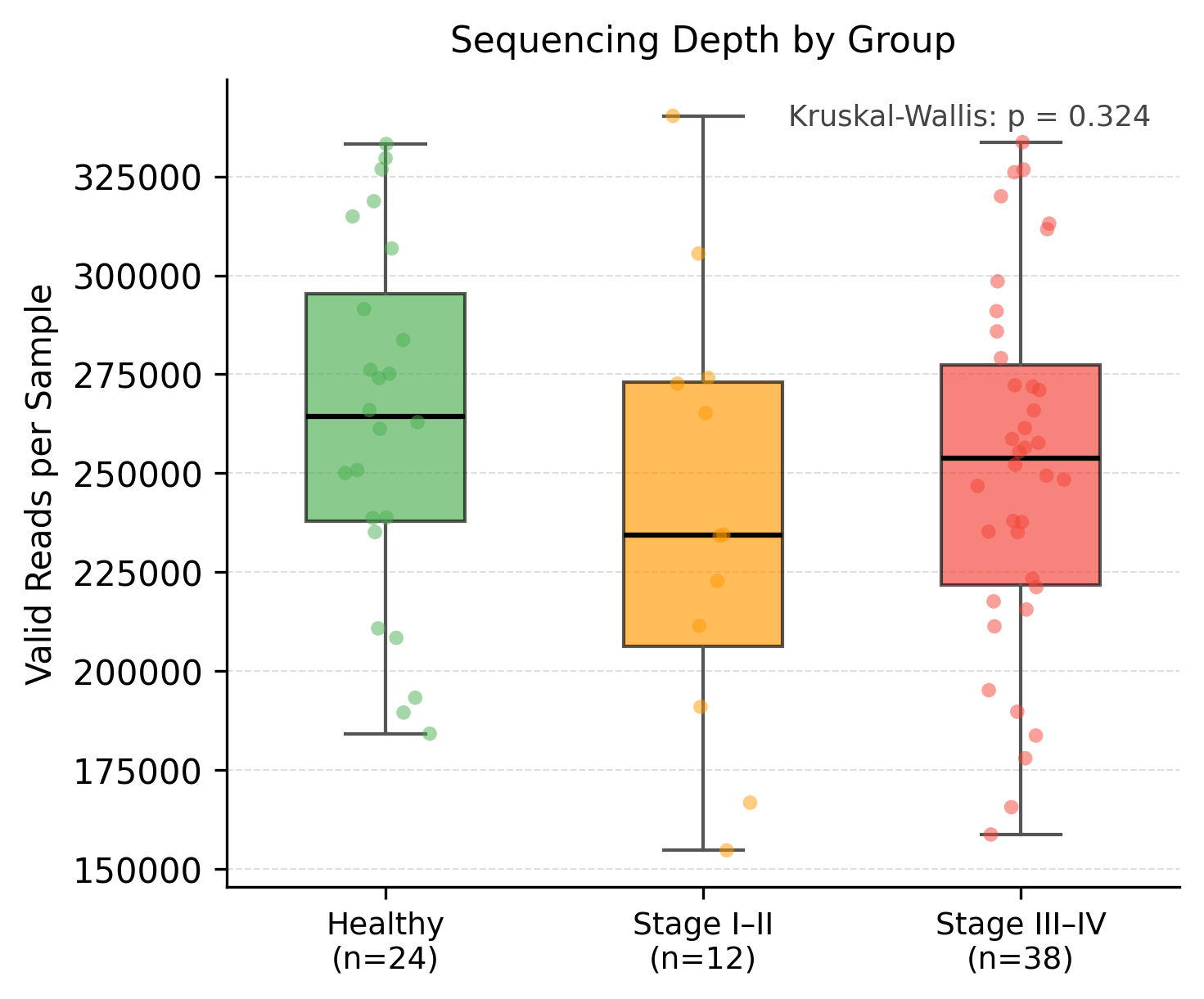

Supplement: Supplementary Figure 1 — Sequencing depth distribution across periodontal status groups. [file Image1.png]
